# Multiple strategies, one mission: mesenchymal stromal cell-based mechanisms of action in osteoarthritis

**DOI:** 10.3389/fcell.2026.1763344

**Published:** 2026-04-20

**Authors:** Maid Junuzović, Antonia Troillet, Janina Burk

**Affiliations:** 1 Physiology and Pathophysiology, Department of Biological Sciences and Pathobiology, University of Veterinary Medicine Vienna, Vienna, Austria; 2 Department for Horses, Faculty of Veterinary Medicine, University of Leipzig, Leipzig, Germany

**Keywords:** chondocytes, ECM remodeling, immunomodulation, macrophage modulation, mesenchymal stromal (stem) cell (MSC), mitochondrial transfer, osteoarthritis, synoviocytes

## Abstract

Osteoarthritis (OA) is a cross-species, multifactorial joint disease characterized by the progressive degeneration of articular cartilage, morphological remodeling of the subchondral bone, and inflammatory and fibrotic changes of the joint capsule. These alterations arise from chronic, often subclinical, inflammatory processes and dysregulated cellular homeostasis, leading to profound shifts in the cellular and extracellular composition of the joint organ. Although the mechanisms driving persistent inflammation are only partially understood, their impact on all joint-associated tissues is well-established. Mesenchymal stromal cells (MSCs) have gained increasing attention as therapeutic candidates for OA due to their immunomodulatory and potentially regenerative capacities. Increasing evidence indicates that MSCs exert their effects predominantly through indirect mechanisms, including paracrine signaling, the release of extracellular vesicles, mitochondrial transfer, and modulation of innate and adaptive immune responses. This review summarizes current insights into how these mechanisms may act within the articular microenvironment to attenuate cartilage degeneration and promote tissue repair in OA. Herein, we consider the effects of MSCs on different cell types and tissues within the joint, and highlight mitochondrial transfer as an emerging mechanism through which MSCs may regenerate and protect them, thereby contributing to the rescue of joint homeostasis.

## 1 Introduction

Mesenchymal stromal cells (MSCs) have been studied extensively for their potential application in cell-based therapies, owing to their many favorable characteristics including ease of isolation and expansion, low immunogenicity and divers e therapeutic mechanisms (Pittenger et al., 2019). Osteoarthritis (OA) was among the first orthopedic conditions for which MSCs were investigated as a therapeutic option. The first study to demonstrate positive effects of MSC application used a caprine model, where OA was unilaterally induced in knee joints by anterior cruciate ligament transection and medial meniscectomy. An intra-articular injection of autologous GFP-labeled bone marrow-derived MSCs followed 6 weeks later. Treated joints showed signs of medial meniscus regeneration, with implanted cells identified in the newly formed tissue. MSCs were also found in the lateral meniscus, synovial capsule and fat pad, but not within the articular cartilage. Reduction of articular cartilage degeneration, osteophytic remodeling, and subchondral sclerosis was documented, although this was rather interpreted as delaying OA progression as cartilage lesions still developed in treated animals (Murphy et al., 2003). Since this promising early study, research activities in this field have dramatically increased, ranging from basic *in vitro* research to controlled, randomized clinical trials.

Numerous trials in human patients have also reported improved cartilage repair and alleviation of OA symptoms following MSC application, as reviewed with an optimistic but careful outlook on MSC for joint regeneration. Summarizing the evidence from different meta-analyses, one review compiled that MSC therapy, particularly with bone marrow and adipose-derived MSC, can provide benefits detectable via MRI and second-look arthroscopy, alongside improvement of clinical symptoms. The authors noted that two meta-analyses revealed that adipose-derived MSCs had more consistent outcomes or outperformed bone marrow MSCs in certain metrics, respectively (Song and Jorgensen, 2022). However, variations in dosage, administration protocols, and follow-up durations limit definitive conclusions, underscoring the need for larger, well-controlled studies with standardized methodologies.

In this line, a systematic review of randomized controlled trials, focusing on their methodology, highlighted two major issues: inconsistent nomenclature and selective outcome reporting (Jones et al., 2019). The authors had analyzed eight randomized clinical trials and found that positive efficacy conclusions might often be overstated, with many studies emphasizing intra-group improvements or inter-group subscore differences when the primary data failed to show significant differences. The authors argue that omitting inter-group comparisons can be misleading and undermine the interpretability of results (Jones et al., 2019). Inevitably, challenges with MSC definition and nomenclature, clinical trial design and reporting bias slow the translational progress, and despite years of research, there is so far only one approved human MSC product for OA (CARTISTEM®, approved for treatment of knee cartilage defects in South Korea).

**FIGURE 1:**
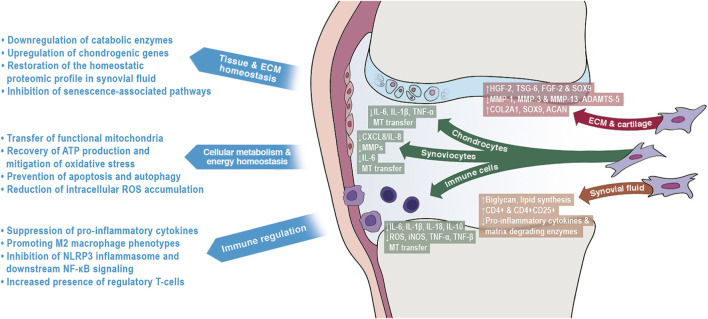
Illustrative overview of the diverse mechanisms through which mesenchymal stromal cells (MSCs) exert their positive effects in osteoarthritis-affected joints.

Another substantial reason for controversial clinical results is our limited understanding of MSCs’ multifaceted, context-sensitive modes of actions in the joint environment (Figure 1). Even seemingly simple decisions such as timing of MSC transplantation or possible anti-inflammatory drug administrations during the therapy strongly impact on the environment into which the MSC are transplanted. This in turn may either promote or decrease certain modes of action and thus, alter therapeutic efficacy.

Supporting this, a recent study shed light on the fate and adaptation of MSCs following intra-articular injection. In a murine collagenase VII–induced OA model, GFP-labeled bone marrow MSCs were injected into affected joints; while most cells did not survive for more than 3 days post-delivery, the remaining MSCs were analyzed. They exhibited increased IL-10 expression and reduced TNF-α levels with transcriptomic data further revealing upregulated extracellular matrix (ECM) organization and cell migration-associated pathways in MSCs retrieved on day 14, whereas MSCs recovered at day 54 showed enhanced expression of immunomodulatory pathways (Ivanovska et al., 2025). These findings suggest that MSCs may contribute to ECM remodeling and cell migration in the early phases of OA, while adopting an immunosuppressive role in later healing phases. Nevertheless, this insight is preliminary as the study could not reflect the naturally occurring OA microenvironment. To fully exploit the therapeutic potential of MSCs, it is crucial to further increase our knowledge of their diverse effects that might become relevant within the environment of the diseased joint.

With MSC engraftment in OA-affected joints at around 3%, and most of the retained cells associating with the synovium, meniscus and fat pad, it became reasonably clear that MSCs did not act through direct cell replacement, as was previously hypothesized, but rather exerted their therapeutic effects in joints through alternative mechanisms (Barry, 2019). This is further supported by the fact that the MSC secretome and/or MSC-derived EVs appear to replicate many of the effects observed with MSCs. MSC-derived extracellular vesicles (EVs) have emerged as a promising tool for cell-free therapies (Mizenko et al., 2024), potentially leading to similar results as direct MSC treatment but with advantages such as ease of storage, lower immunogenicity and improved agent standardization. A comprehensive understanding of these alternative mechanisms requires consideration of the joint as an integrated organ system rather than focusing solely on the articular cartilage, as increasingly addressed in current research (Linde et al., 2025). Ultimately, advancing our knowledge on OA pathophysiology and MSC biology will be essential to elucidate the full complexity of disease processes and therapeutic responses.

## 2 MSC sources: spoilt for choice?

Aiming to promote standardized and scientifically accurate terminology, the International Society for Cell and Gene Therapy (ISCT) issued a position statement for the clarification of MSC nomenclature in 2006. It states that (1) MSC must be plastic-adherent when maintained in standard culture conditions, (2) express CD105, CD73 and CD90, (3) lack the expression of CD45, CD34, CD14 or CD11b, CD79α or CD19 and HLA-DR surface molecules and (4) must be able to differentiate into osteoblasts, adipocytes and chondroblasts *in vitro* (Dominici et al., 2006). In 2019, the ISCT issued an additional position statement recommending the acronym “MSC” to be accompanied with a clarification of whether it refers to “mesenchymal stromal cells” or “mesenchymal stem cells” depending on the context, along with the tissue origin (Viswanathan et al., 2019).

In recent years, the term MSCs has increasingly been described as “medicinal signaling cells,” reflecting the growing recognition of their paracrine signaling, extracellular vesicle production, and mitochondrial transfer as key mediators of their therapeutic effects (Caplan, 2017; Fontaine et al., 2016; Caplan and Correa, 2011).

Although MSCs can be obtained from virtually any vascularized tissue in the body, the most commonly used sources of MSC have historically been bone marrow and adipose tissue. Bone marrow has been the longest utilized source of MSCs. However, the procurement itself is relatively invasive. In contrast, adipose tissue is abundant, easily accessible throughout the body, and often available as a byproduct of surgical procedures or liposuction. With particular relevance to OA therapeutic approaches, the synovial membrane (De Bari et al., 2001) and synovial fluid (SF) (Jones et al., 2004) are also well-documented sources of MSCs.

Regardless of the tissue of origin, all MSCs share certain similarities, particularly in terms of their multipotency and immunomodulation capabilities. However, variations observed in their properties can result in a certain degree of heterogeneity. This can be attributed to multiple factors, including but not limited to individual donors and tissue sources, cell isolation techniques, culture conditions, cryoprotective and thawing protocols (Nicolay et al., 2015; Fong et al., 2016; Yin et al., 2019), and.

Differences between tissue sources may influence the suitability of MSCs for specific clinical indications, as suggested in several studies comparing the differentiation capacity of MSCs from different tissues under defined conditions. An early study found that bone marrow, synovial and periosteum-derived MSC exhibited the highest osteogenesis rate, while synovium- and adipose tissue-derived MSC showed greater adipogenic potential than periosteum- and muscle-derived cells (Sakaguchi et al., 2005). Studies investigating the chondrogenic potential of human MSCs derived from different tissues found that synovial fluid-derived MSCs had greater chondrogenic potential compared to MSCs from bone marrow, periosteum, skeletal muscle and adipose tissue (Sakaguchi et al., 2005), and infrapatellar fat pad-derived MSCs had greater chondrogenic differentiation capacity than MSCs from bone marrow and subcutaneous fat (Garcia et al., 2016). Corresponding findings have also been reported in other species, with ovine synovial fluid–derived MSCs exhibiting higher chondrogenic potential than bone marrow–derived MSCs (Burk et al., 2017) and similar observations in equine tissues (Fülber et al., 2016).

It is particularly interesting that MSC from joint-associated tissues, namely synovial fluid or membrane and the infrapatellar fat pad, exhibited the highest therapeutic chondrogenic potential in several studies (Sakaguchi et al., 2005; Garcia et al., 2016; Fülber et al., 2016; Burk et al., 2017). Although chondrogenic differentiation potential alone may not be sufficient to qualify the MSCs for joint regeneration, these findings still suggest that niches within the joint harbor MSCs with superior potential for regenerating this specific environment. This supports the existence of an environmental niche memory concept (Rendra et al., 2020) which may shape MSC behavior through distinct paracrine, immunomodulatory and/or metabolic mechanisms and play a role in selecting the most applicable MSC tissue sources for specific clinical applications.

Reinforcing the idea that MSC sources influence their therapeutic efficacy in OA, an *in vivo* study using a collagenase II-induced rat OA model compared intra-articular injections of adipose-derived MSCs and synovial membrane-derived MSCs. While the cells from both sources showed beneficial effects in the defined pathological criteria, synovial membrane MSCs entailed better results than their counterparts from adipose tissue (Zare et al., 2020).

Nevertheless, the response to MSCs derived from different adipose tissue sources including the Hoffa fat pad varied more between chondrocytes from different OA donors than between adipose tissue types (Manferdini et al., 2013). Therefore, while careful consideration of the MSC source is indicated, this may still have less impact on treatment outcome than factors associated with the recipient and joint environment.

## 3 How MSC navigate to inflamed tissues: keys to homing and attachment

The ability of MSC to migrate to sites of injury or inflammation is a key factor of their therapeutic effects. Commonly referred to as homing, this phenomenon is investigated for its potential to enable exogenously administered MSC to migrate to injured tissues (Caplan, 2009; Singer and Caplan, 2011; Le Blanc and Mougiakakos, 2012; Bronckaers et al., 2014).

Several authors have reported that only a relatively low percentage of systemically administered MSCs reach their target tissue (Devine et al., 2003; Barbash et al., 2003; Kraitchman et al., 2005). Contributing factors include the inherently low expression of homing molecules such as CXCR4 in MSCs (Wynn et al., 2004; Lüttichau et al., 2005), as well as further reduction of their expression levels during *in vitro* expansion (Rombouts and Ploemacher, 2003; Honczarenko et al., 2006). Additionally, intravenously administered MSCs can become trapped in small pulmonary capillaries (Scarfe et al., 2018), potentially hindering homing efficiency and leading to adverse side-effects including pulmonary (Boltze et al., 2015) or cerebral microembolisms (Cui et al., 2015). Although this is often described as a transient first-pass effect (Fischer et al., 2009), the relevance of the phenomenon is supported by studies showing that vasodilators and anticoagulants reduce lung trapping and enhance MSC homing (Gao et al., 2001; Yukawa et al., 2012). However, for OA therapies, this issue is not of major relevance as direct intra-articular injection is a feasible delivery route and homing only needs to occur within the joint.

### 3.1 Molecular and cellular factors associated with homing in OA

Under physiological conditions, joint-associated tissues contain specialized MSC niches that provide a microenvironment for MSC to remain in a quiescent, undifferentiated state (Zhang et al., 2020). In OA, these microenvironments are altered, becoming enriched with various factors that can initiate homing. Many chemotactic factors, such as IL-6, CCL2 or CXCL12/SDF-1, are upregulated in OA-affected SF, synovial membrane and cartilage (Smith et al., 1997; Furman et al., 2015; Watt et al., 2016; Fernandez-Pernas et al., 2017; Hou et al., 2020), with inflammatory cells, chondrocytes, and even MSCs themselves being potential sources. Additionally, cell lysates, fragments of the ECM and high mobility group box chromosomal protein 1 (Vorotnikova et al., 2010; Seol et al., 2012; Yu et al., 2014; Lin et al., 2016) function as recruiting signals for MSCs. Functionally, exposure to trauma-associated factors in conditioned medium *in vitro* led to improvements in directed cell migration of chondrogenic stem/progenitor cells, which possess multipotent differentiation capacities and similar surface marker expression profiles as MSCs (Riegger et al., 2018).

Studying the effects of IL-1β on chondrocytes from OA-affected human articular cartilage and same-donor bone marrow- and adipose-derived MSCs revealed that all cell types responded with changes in pro- and anti-inflammatory mediator secretion. However, unlike MSCs, chondrocytes showed a higher basal secretion of growth factors and increases in almost all immunomodulatory cytokines in response to IL-1β (De Luca et al., 2019), highlighting them as a source of chemoattractants for MSCs during OA. Nevertheless, it remains to be acknowledged that as already pointed out above, MSCs do not readily attach to the cartilage but rather adhere to other tissues within the joint (Murphy et al., 2003). Therefore, chemoattractants from chondrocytes alone are not sufficient for successful MSC integration, raising the question of what differentiates the tissues to which MSCs adhere from the articular cartilage in terms of homing and attachment signals. Several aspects apply, namely ECM-related factors and resident cell-related factors, which might act in concert.

### 3.2 ECM composition and MSC interaction with immune cells

Hyaline cartilage ECM is dense and mainly composed of collagen II and aggrecan, as further described below. While binding sites and binding affinity of collagen I to stromal cell surface receptors such as integrins are well-characterized (Di Lullo et al., 2002; Heller et al., 2004), specific literature on collagen II binding is scarce. Thus, MSCs are expected to bind readily to collagen I–rich connective tissues, such as adipose tissue stroma and the intimal synovial layer of the joint capsule; whether collagen II in hyaline articular cartilage exhibits a similar binding affinity for MSCs has not been fully elucidated. Furthermore, articular cartilage ECM and SF hyaluronan provide a negatively charged hydrophilic environment, and negative charge was shown to impact on cell adhesion and chondrogenesis (Cowman et al., 2015; Yang et al., 2020). Moreover, the structural organization of the ECM also influences MSC localization. Articular cartilage is densely organized, and we previously observed that even in collagen I–rich tendons, regions with tightly packed fibers were less efficiently populated by MSCs than areas with more loosely arranged matrix (Burk et al., 2016).

Besides the distinct cellular and extracellular matrix composition of the cartilage, the joint as an organ comprises additional specialized tissues containing distinct cell populations, which might steer MSCs within the joint cavity. Namely, the synovial lining of the intimal layer of the joint capsule comprises highly specialized and dynamic cell populations, which can be distinguished as macrophage-like immune cells or fibroblast-like stromal cells (Smith, 2011). Conditioned medium from OA-affected synovium was shown to enhance MSC migration *in vitro*. Interestingly, treatment with triamcinolone acetonide further amplified this effect by reducing pro-inflammatory cytokines such as TNF-α, IL-1β, and IL-6, while promoting markers associated with anti-inflammatory macrophages. Moreover, conditioned medium from anti-inflammatory and tissue-regenerative M(IL-4) and M(IL-10) triamcinolone-treated macrophages significantly improved MSC migration. In contrast, conditioned media from pro-inflammatory M(IFNγ+TNFα) macrophages or synovial fibroblasts had no significant impact. This suggested that modulating inflammation with triamcinolone enhances the migration-promoting activity of anti-inflammatory macrophages, which may be valuable for OA therapeutic strategies (Wesdorp et al., 2022). Furthermore, these findings indicate that the presence of anti-inflammatory macrophages and other immune cells may be not only beneficial but potentially even necessary for effective MSC homing and recruitment.

Moreover, MSCs do not only respond to chemotactic signals but can also enhance the homing of other cells, hinting at possible reciprocal mechanisms. Co-transplantation of human umbilical cord MSCs with umbilical cord blood mononuclear cells in a NOD/SCID mouse model led to increased expression of VEGF-A, OPN, and SDF-1α in the bone marrow and suggested that MSCs promote engraftment of cord blood mononuclear cells via providing a hematopoetic microenvironment (Huang et al., 2021). Comparable results were observed with bone marrow cells transplanted in myeloablated mice with increased COL4A1 and COL4A2 expression, where co-transplantation with tonsil-derived MSCs led to improved bone marrow cellularity. This appeared to be mediated by increased matrix metalloproteinase (MMP)-3 secretion and resulting collagen IV breakdown by the MSCs, suggesting they facilitated cell migration by reducing physical barriers for chemotaxis (Lee et al., 2021). Comparable mechanisms may also steer the homing of MSCs within OA joints; however, direct experimental evidence in this context remains elusive.

### 3.3 Endogenous mobilization of MSCs and enhancement strategies

Homing signals cannot only be employed to guide exogenously administered MSCs. A study using human synovium MSCs embedded in a hydrogel composed of Matrigel and type I collagen demonstrated that platelet-derived growth factor-BB (PDGF-BB) effectively promotes MSC mobilization. This was confirmed *in vivo*, where intra-articular injection of PDGF-BB in rat knees increased the number of colony-forming MSC-like cells in the synovial fluid (Endo et al., 2021). This suggests that cytokine-driven endogenous mobilization of resident synovium-derived MSCs may serve as an alternative to exogenous cell delivery strategies; however, its efficacy remains to be investigated. In a clinical setting, a proof-of-concept study evaluated an arthroscopically applied mechanical stem cell-mobilizing (STEM) device designed to recruit endogenous synovium-derived MSCs. In patients treated with microfracture, colony-forming unit–fibroblast measurements taken before irrigation, after irrigation, and following STEM use demonstrated that irrigation nearly eliminated MSCs from the synovial fluid, whereas the subsequent STEM treatment increased their number by about 40-fold (Altaie et al., 2024). However, it should be noted that the patient cohort in this study was small, and further research is required to evaluate this device as a potential therapeutic strategy for early OA before firm conclusions can be drawn.

Despite challenges such as low homing efficiency and pulmonary trapping following systemic delivery, the OA joint provides a chemotactically active microenvironment, rich with cytokines, ECM fragments, and damage-associated molecules that may attract both endogenous and exogenously administered MSCs. Their recruitment may be facilitated by upregulated factors such as IL-6, CCL2, and SDF-1, which are highly expressed in OA-affected synovium and cartilage (Smith et al., 1997; Furman et al., 2015; Watt et al., 2016; Fernandez-Pernas et al., 2017; Hou et al., 2020).

Strategies to improve MSC homing, with specific relevance to OA therapies focus mainly on *in vitro* priming, modification of the target tissue e.g. using ultrasound, and MSC (surface) engineering (Ullah et al., 2019). In particular, MSC engineering approaches have focused on overexpressing migration-related genes, including CCR-2 (Zheng et al., 2019), CXCL-9 (Li M. et al., 2021), c-Met (Wang et al., 2017) and CXCR-4 (Du et al., 2013). Notably, MSC attachment appears to occur mostly in niches characterized by non-cartilage connective tissue ECM, possibly mediated by resident MSC- and macrophage-like cells. Although not directly leading to cartilage replacement, this still allows for multiple effects on the joint environment.

## 4 The synovial compartment: a multifaceted target

The synovial niche is not only important as a major destination of MSC homing within the joint, but also due to its manifold contributions to joint physiology and pathophysiology. Diarthrodial (synovial) joints, which are the most commonly affected by osteoarthritis, feature a distinctly multilayered joint capsule, hyaline articular cartilage covering the articulating epiphyseal bone surfaces, and synovial fluid filling the joint cavity. The histomorphological organization of the joint capsule comprises an inner stratum synoviale and an outer stratum fibrosum, the latter merging seamlessly with the surrounding connective and supportive tissues of the periarticular region. The stratum synoviale is of particular relevance to joint biology and pathology, and is subdivided into the synovial intima and the subintimal layer.

The initima harbours two distinct types of synoviocytes. Macrophage-like, or type A synoviocytes (referred to as synovial macrophages) are mainly involved in phagocytic functions und immunoregulation. Fibroblast-like or type B synoviocytes (referred to as synovial fibroblasts) have secretory abilities (Castrogiovanni et al., 2019) and produce hyaluronan and lubricin (Chang et al., 2010), major constituents of the synovial fluid. Both THY1^+^ (CD90^+^) and THY1^-^ (CD90^−^) synovial fibroblasts share several phenotypic characteristics with MSCs (Damerau et al., 2022).

In the subintimal layer, the structural organization of the joint capsule changes markedly. This compartment is comparatively cell-poor yet highly vascularized, and consists predominantly of loosely organized connective tissue with sparsely distributed synovial fibroblasts and macrophages, adipocytes, and only minimal numbers of infiltrating inflammatory cells (Edwards, 1994; Smith, 2011).

Synovial fluid is a viscous, non-Newtonian fluid composed primarily of hyaluronic acid, lubricin, and interstitial fluid. It fills the cavities of synovial joints and plays a critical role in nutrient transport to avascular joint tissues, with the nutrition of articular cartilage being primarily supplied through diffusion from the SF (Cowman et al., 2015; Brody, 2015). Moreover, SF harbors a range of immune and stromal cells and, as described above, serves as a source of MSCs with high chondrogenic potential. Consequently, it acts not merely as a passive reservoir but also as an active communication interface that mediates cellular and molecular signaling within the joint.

### 4.1 Synoviocyte and macrophage pathophysiology

Highlighting the contribution of synovial cells to OA pathogenesis, single-cell RNA sequencing has provided valuable insights into the molecular crosstalk between synovium and cartilage. By profiling synoviocytes and chondrocytes from matched OA tissues, 12 distinct synovial cell types and 7 chondrocyte phenotypes were identified (Chou et al., 2020). Damaged cartilage was enriched for prefibro-, fibro-, regulatory, reparative, and prehypertrophic chondrocytes and a total of 61 cytokines and growth factors were predicted to regulate these chondrocyte phenotypes. Of these, 55% were produced by synovial cells, with 39% being exclusive to synoviocytes. The production of IL-1β was primarily attributed to inflammatory macrophages and dendritic cells within the heterogeneous synoviocyte population, characterized by co-expression of HLA-DQA1, HLA-DQA2, OLR1, or TLR2 (Chou et al., 2020). This indicates that the synovium does not have a merely passive role in OA but actively influences OA progression by modulating cytokine and growth factor release.

Despite their relevance, the therapeutic effects of MSCs on synoviocytes in OA remain underexplored, as most studies in that context focused on rheumatoid arthritis (Xu et al., 2018; Ding et al., 2022; Bok et al., 2025). However, it has been demonstrated that human adipose-derived MSCs exert anti-inflammatory effects on both chondrocytes and synoviocytes from OA patients when co-cultured in transwells. MSCs induced a significant reduction in the expression and release of several pro-inflammatory cytokines including IL-6 and CXCL8/IL-8, at which the COX2/PGE2 pathway appeared to play a role. However, the response to the MSCs varied between OA tissue donors (Manferdini et al., 2013). Complementing these findings, a canine *in vitro* model revealed that adipose MCSs were capable to induce downregulation of matrix remodeling MMPs in LPS-challenged synoviocytes (Oh et al., 2021).

While the above-mentioned studies on synoviocytes in OA did not discriminate between the two major phenotypes of synoviocytes, both synovial fibroblasts and macrophages are likely to be relevant targets of MSC-based OA therapies.

Activated synovial fibroblasts contribute to OA by secreting pro-inflammatory cytokines such as IL-6 (Brennan and McInnes, 2008) and undergoing senescence-associated changes (Malaise et al., 2021). They also produce MMPs and VCAM-1, which can stimulate chondrocytes to release catabolic enzymes (Abramson and Attur, 2009), thereby exacerbating the degradation of articular cartilage (Bartok and Firestein, 2010; Müller-Ladner et al., 2007). Vice versa, the presence of cartilage wear particles, debris of the degradation of articular cartilage in OA, has been shown to increase release levels of nitric oxide (NO), IL-6, IL-8 and several MMPs, specifically MMP-9, -10 and -13, from synovial fibroblasts (Estell et al., 2019). Moreover, THY1^+^ synovial fibroblasts in OA were shown to undergo metabolic reprogramming towards glycolysis, which contributes to cartilage damage (Damerau et al., 2022). Yet unfortunately, despite their role in OA pathogenesis, no insights on the effects of MSC treatment on synovial fibroblasts are available from the literature so far. This knowledge gap could be critical, considering that synovial fibroblasts have an important role in linking immune activation and tissue remodeling processes.

Under resting conditions, tissue-resident macrophages, together with fibroblasts, form an immunological barrier isolating the joint cavity (Smith, 2011; Culemann et al., 2019). However, these cells are not merely passive barriers but actively contribute to local tissue homeostasis (Mosser et al., 2021). Research in the past few years has focused on the involvement of synovium-derived macrophages in OA pathogenesis, demonstrating that they influence the severity of OA (Zhang et al., 2018; Park et al., 2020). Providing further evidence, the experimental depletion of the highly organized population of CX3CR1+ macrophages within the murine synovial lining resulted in barrier breakdown and uncontrolled joint inflammation (Culemann et al., 2019). This suggests that lining-macrophages play an important role in joint homeostasis by actively modulating inflammation, challenging our traditional understanding of synovial anatomy and function.

Macrophages are a heterogeneous cell population capable of rapidly adopting distinct polarization states in response to their local environment (Liu et al., 2015). Based on their (dynamic) polarization state, they are often subdivided into classically activated (M1; CD86^+^) and alternatively activated (M2; CD206+) macrophages. Pro-inflammatory cytokines are more likely to stimulate M1 macrophage polarization, while M2 macrophage polarization is influenced more by anti-inflammatory cytokines (Murray, 2017). However, different combinations of cytokines can also result in different macrophage phenotypes, as was shown in a study where different combinations of IL-4, IL-10, IL-13, and TGF-β were used to stimulate M2 macrophage polarization and demonstrated that each of these cytokine combinations resulted in slightly different macrophage phenotypes (Mia et al., 2014).

The M1 macrophage-associated cytokines IL-6, IL-1β, TNF-α and oncostatin M can promote destructive and inflammatory processes in chondrocytes, including downregulation of collagen type II and aggrecan synthesis (Fahy et al., 2014). They also increase the levels of catabolic enzymes such as MMPs (Chi et al., 2022) and support the recruitment of immune cells to joints (Tu et al., 2018). In contrast, M2 macrophages are hypothesized to play a significant role in tissue regeneration and remodeling. By secreting anti-inflammatory cytokines such as IL-10 and TGF-β, they regulate the immune responses and promote tissue repair and angiogenesis (Liu et al., 2020). Additionally, M2 macrophages release growth factors and mediators that stimulate fibroblasts to synthesize ECM components and support vascularization, ensuring adequate oxygen and nutrient supply to regenerating tissues (Sutherland et al., 2023).

In an equine model, cells expressing CD14, CD86, and CD206, markers characteristic of distinct synovial macrophage subtypes, were identified in synovial tissue. Using CD86 to identify the proinflammatory M1 phenotype, it was evident that joints with more severe osteoarthritis and higher levels of proinflammatory cytokines and chemokines showed a shift toward M1 polarization. These findings underline the role of synovial macrophage polarization in local inflammation and tissue degradation, suggesting that targeting macrophages could be a potential therapeutic strategy (Menarim et al., 2020).

### 4.2 Macrophage modulation by MSCs and their products

MSCs have been shown to influence macrophage polarization in numerous studies, although most were not focused on the OA environment or dealing with synovial macrophages. Co-culturing lipopolysaccharide (LPS)-stimulated RAW264.7 mouse peritoneal macrophages and MSCs revealed that MSC diminished the levels of pro-inflammatory cytokines in treated RAW264.7 cells, namely IL-6, IL-1β, and iNOS. They also skewed the macrophages towards an M2 polarization-like state via a paracrine mechanism, which was later identified to involve TGF-β secreted by MSCs (Liu et al., 2019). Similar results were observed in a study using murine lung organoids derived. Lung tissue was co-cultured with alveolar and interstitial macrophages using matrigel to construct an *in vitro* lung organoid–immune cell system. Treatment of the organoids with LPS led to significant upregulation of chemokines and inflammatory cytokines, namely CCL3, CCL4, CCL5, CXCL1, CXCL2, IL-1β and TNF-α, as well as enhanced macrophage chemotaxis towards the organoids. However, when co-cultured with MSCs, the LPS-induced chemotactic aggregation of macrophages was significantly reduced. Expression of inflammatory chemokines was suppressed and ROS production in alveolar macrophages inhibited (Zhu et al., 2024).

Not only MSCs, but also their EVs were repeatedly shown to be capable of macrophage modulation. Among the various types of MSC-derived EVs, exosomes—small membrane-bound vesicles—have been particularly well-studied for their ability to influence macrophage polarization. Several studies have also explored how priming during exosome production may enhance their immunomodulatory effects. Exosomes isolated from human bone marrow MSCs, both from untreated MSCs and MSCs treated with melatonin, were shown to significantly reduce IL-1β and TNF-α secretion in treated RAW264.7 macrophages while increasing IL-10 levels, indicating a potential shift towards M2 polarization. This was further supported using streptozotocin-induced diabetic rats, where melatonin treatment improved the M2/M1 macrophage polarization ratio by activating the PTEN/AKT signaling pathway. Improved wound healing, increased collagen synthesis, and enhanced angiogenesis were also observed in the treated animals (Liu et al., 2020).

Using preconditioning strategies based on inflammatory licensing, exosomes from human umbilical cord MSCs primed with IL-1β promoted a M1/M2 macrophage shift, shown by an increased CD206/CD86 ratio. Similar but less pronounced effects were seen with exosomes from non-primed MSCs (Chen et al., 2025). In the same line, macrophages stimulated with exosomes from human gingival tissue-derived MSCs exhibited a notable increase in CD206 expression (Nakao et al., 2021). Exosomes obtained with TNF-α preconditioning tended to have a stronger impact than without preconditioning, further increasing the proportion of CD206^+^ macrophages as compared to control exosomes. However, other preconditioning strategies with LPS, IFN- γ or acetylsalicylic acid did not improve the yield of CD206^+^ macrophages. Exosomes obtained under TNF-α preconditioning also elevated IL-10 expression while reducing pro-inflammatory mediators such as IL-1β, TNF-α and iNOS. A complementary *in vivo* mouse model showed that injections of exosomes enhanced wound healing, with TNF-α-preconditioned exosomes accelerating the process compared to control exosomes (Nakao et al., 2021).

### 4.3 Macrophage modulation in OA environments

With particular focus on OA, in a mouse model where OA was induced by surgically removing the medial meniscus and anterior cruciate ligament, human umbilical cord MSC-derived EVs lowered the expression levels of IL-1β and IL-18 and achieved anti-inflammatory effects by inhibiting the NLRP3 inflammasome through downregulation of METTL3 expression in macrophages, facilitating a shift toward the M2 phenotype (Zhou et al., 2022). Furthermore, building on previous findings that culturing human bone marrow MSCs in 3D gelatin microgels avoided core hypoxia and enhanced therapeutic efficacy compared to conventional 2D equivalents (Wang et al., 2022), the same research group employed this 3D culture system to produce MSC-derived exosomes and analyzed their effects on macrophage polarization and OA progression (Yan et al., 2024). Exosomes from 3D cultures more effectively induced M2 polarization compared to exosomes from 2D cultures, primarily through increased expression of miR-365a-5p. , which inhibited the TLR2/Myd88/NF-κB axis in macrophages, enhancing their anti-inflammatory phenotype. These findings support the notion that priming in 3D cultures enhances the immunomodulatory and regenerative effects of MSC-derived exosomes in OA therapy (Yan et al., 2024).

Comparing the effects of human umbilical cord MSCs and their small EVs in an anterior cruciate ligament transection rat model, with respect to immunomodulation and macrophage polarization, CD14 and IL1β immunostaining of joint sections was reduced and CD206 and IL10 staining increased in both treatment groups, indicating a similar effect of MSCs and EVs on macrophage modulation (Tang et al., 2021).

MSC-derived ECM can also modulate macrophages. An MSC-ECM-functionalized hydrogel, combining gelatin methacryloyl with ECM derived from MSCs treated with IL-6, suppressed M1 but promoted M2 macrophage polarization and induced macrophage metabolic reprogramming. When the supernatant from the reprogrammed macrophages was added to chondrocytes under a state of homeostatic imbalance, a reduction in MMP13 expression was observed, providing proof of functional changes with benefit in OA. *In vivo*, using a rat model with destabilization of the medial meniscus, MSC-ECM treatment suppressed cartilage degeneration and inhibited synovial inflammation (Chen et al., 2024).

Overall, there is strong evidence that MSCs or their products are capable of modulating inflammation via macrophages. This highlights the interaction between MSCs and macrophages as a significant component in modulating the immune response in OA-affected joints. As reviewed in detail recently (Araya-Sapag et al., 2025), this approach holds great promise for OA therapies, although more in-depth insight on the interaction of MSCs and synovial compartment macrophages is still required.

### 4.4 Effects of MSCs on synovial fluid composition

Chondrocyte viability and functionality depend heavily on the SF, which supplies essential nutrients to chondrocytes via diffusion and serves as a medium of communication between the joint compartments. In OA joints, alterations in SF cellular and ECM composition could compromise cartilage homeostasis and accelerate degeneration.

Many studies analyzed the effects of SF from healthy and OA-affected joints on MSCs; however, research into alterations of the SF itself following intra-articular MSC application is scarce, although the SF is easily accessible in larger animals. One study, using a sheep model, analyzed the immune cells in SF following intra-articular administration of xenogeneic human umbilical cord MSCs and hyaluronic acid (HA) in healthy knee joints and knee joints with OA induced by medial meniscal release surgery. SF samples and synovial membrane biopsies were collected at regular intervals over 13 weeks. A significant but transient increase of CD4^+^ and CD4^+^CD25^+^ cells was observed in treated animals, irrespective of OA, suggesting an increased presence of regulatory T cells. Neither alterations of CD8^+^ or MHCII+ cell percentages nor long-term alterations to the overall lymphocyte profile in the SF could be observed, and no significant differences in iNOS+ synovial lining cell percentages, abnormal villi and total synovium score were found (Lamers et al., 2020).

It is important to consider species-specific immunological features when interpreting these results. For instance, γ/δ T cells make up an inconsequential amount of SF and synovial membrane infiltrates in humans with OA (Andreu et al., 1991). These cells, however, could not be assessed in the study, as their small ruminant counterpart is different from the human, with a higher proportion and increased diversity of receptors on small ruminant circulating γ/δ T cells (Plattner and Hostetter, 2011; Holderness et al., 2013).

In a second study using a similar xenogeneic sheep model, SF was collected 12 weeks after injection of human umbilical cord MSCs and subjected to proteomic analysis. Nineteen SF proteins were differentially abundant in treated animals, most notably biglycan - a leucine-rich proteoglycan found in cartilage ECM. Lipid synthesis and immune cell migration pathways were likely to be activated in treated sheep, whereas tissue damage-, senescence-, inflammation-, vascular permeability- and necrosis-associated pathways were likely to be inhibited (Wright et al., 2023).

Two other studies, although not focusing extensively on SF analyses, revealed that repeated injections of MSC conditioned medium or treatment with exosomes decreased pro-inflammatory mediators and matrix-degrading enzymes in the SF, the former in dogs with naturally occurring OA (Huňáková et al., 2020) and the latter in surgically as well as enzymatically induced rat OA models (Yang et al., 2024).

Although only few studies give insights into this topic so far, their findings indicate that intra-articular MSC (or MSC derivative) application helps to restore the SF microenvironment by modulating its proteomic and immunological profile. The observed immunomodulatory effects in SF further support the role of MSCs in reshaping the joint environment to better support cartilage maintenance and repair.

## 5 The articular cartilage: dampening the damage

Degeneration of articular cartilage represents a cardinal feature of osteoarthritis. Articular cartilage, the specialized connective tissue covering the epiphyseal surfaces of diarthrodial joints, provides a low-friction interface critical for joint function. This hyaline cartilage is composed predominantly of chondrocytes embedded within a highly organized extracellular matrix (ECM), which is rich in type II collagen and proteoglycans, thereby ensuring tissue resilience, load distribution, and structural integrity. As OA progresses, inflammatory processes contribute to the degradation of articular cartilage, ultimately leading to subchondral bone exposure (Armiento et al., 2019).

With no direct blood or lymphatic supply, the regenerative capacity of cartilage in joints is inherently limited (Im, 2018). Although rudimentary repair tissue can form *de novo*, it integrates poorly with the surrounding native tissue (Armiento et al., 2019) and lacks the comparable biomechanical properties, leading to compromised clinical function (DiBartola et al., 2016). A case series with second-look arthroscopic evaluation, at an average of 2 years after the initial osteotomy and without cartilage regeneration strategies, reported fibrocartilage in 92% of medial femoral condyle lesions, while hyaline-like maturation of regenerated cartilage was found in only 4% (Jung et al., 2014).

Hyaline cartilage is the most clinically relevant type of cartilage, being in the focus of intra-articular MSC injection studies (Wei and Bao, 2022). Adult hyaline cartilage is classically stratified into four distinct zones-superficial, middle, deep, and calcified-characterized by differences in chondrocyte morphology, density, and the orientation of collagen fibers within the extracellular matrix (Brody, 2015). The ECM of cartilage is synthesized by chondrocytes, the sole terminally differentiated cells present, with the composition of the matrix varying across the four different zones (Armiento et al., 2019). It is rich in ground substance, primarily glycosaminoglycans (GAGs) and collagen fibers, predominantly type II. By linking with core proteins, GAGs form proteoglycans, with aggrecan being the largest and the most abundant among them (Roughley and Lee, 1994). The low friction surface of hyaline cartilage relies on the physiological joint surface morphology and synovial fluid (Janicka et al., 2019). Aging, but also injury, can damage to the outer surface of hyaline cartilage, leading to increased friction, contributing to the onset of OA (Li Y. et al., 2021).

Irregular collagen expression during tissue repair can result in the formation of fibrocartilage and/or hypertrophic cartilage in recovering joints. Fibrocartilage can be considered a transitional tissue between hyaline cartilage and regular connective tissue, such as tendons and ligaments (Benjamin and Ralphs, 2004). In contrast to hyaline cartilage, it contains high levels of type I collagen and a smaller amount of ground substance. Hypertrophic cartilage is the natural transitional tissue during bone growth, but is considered an undesirable form of cartilage during *in vitro* cell differentiation and joint repair. Characterized by non-dividing enlarged chondrocytes that accumulate glycogen, lipids and alkaline phosphatase, chondrocyte hypertrophy occurs at the expense of the ECM production. The production of short-chain type X collagen fibers can be observed as a consequence (Armiento et al., 2019).

### 5.1 De novo ECM expression

Given its many roles, including structural support, compartmentalizing tissue and facilitating intercellular communication, the composition and health of the ECM are critical for maintaining cartilage homeostasis. Effective OA therapies would ideally stimulate the synthesis of collagen type II to regenerate the native biomechanical properties of articular cartilage. Regrettably, its production is primarily limited to cartilage development, with no evidence of significant turnover in adult or diseased tissues (Armiento et al., 2019).

Nevertheless, several studies showed that MSCs enhance the gene expression of COL2A1, encoding the alpha-1 chain of collagen II, and other chondrogenic genes under specific conditions. *In vitro*, upregulation of COL2A1 was observed in studies where human chondrocytes from OA-affected joints were co-cultured with human bone marrow MSCs (Zhang et al., 2016), as well as in rat chondrocytes after being treated with H_2_O_2_ and then co-cultured with adipose-derived MSCs (Ahmed et al., 2014). In human synovial fluid MSCs isolated from OA-affected joints, COL2A1 expression was increased upon treatment with TGF-β1 under normoxia (20% O_2_). Under hypoxia (5% O_2_), COL2A1 expression increased even further (Neybecker et al., 2018), possibly because this level of hypoxia better mimics the low oxygen tension of native articular cartilage. Despite these promising *in vitro* results, the same study could not find corresponding benefits *in vivo* when using a surgical rat model (Neybecker et al., 2018).

However, applying synovial membrane-derived MSCs from a human OA patient in a rat model with iodoacetate-induced OA showed higher COL2A1 expression levels in treated joints compared to the controls. This was accompanied by radiological and histopathological evaluation showing ameliorated cartilage damage in the MSC-treated group (Rahmadian et al., 2024). Similar evidence comes from a study where a cocktail of hyaluronic acid and human amniotic MSCs was administered into knee joints in a rat iodoacetate OA model. Analysis of cartilage after treatment confirmed the positive effect of MSCs, with significantly higher COL2A1 expression levels in the cartilage of the treated group (Wang et al., 2020). In different studies, similar trends were observed with other chondrogenic genes, namely ACAN and SOX9 (Zhang et al., 2016; Neybecker et al., 2018). ACAN encodes aggrecan (Ahmed et al., 2024), while SOX9 is a key transcription factor in multiple phases of chondrogenesis (Lefebvre and Dvir-Ginzberg, 2017), thus in turn promotes transcription of collagen II and aggrecan genes.

Overall, there is evidence that MSCs can express COL2A1 and enhance the expression of COL2A1 alongside other chondrogenic genes, both *in vitro* and *in vivo*. Yet given the inherently limited collagen II production in adult tissues, alongside with *in vivo* results failing to demonstrate sufficient *de novo* synthesis of hyaline cartilage, the benefits of increased ECM gene expression alone may not be sufficient. Rather, it appears crucial to additionally protect the existing cartilage from degradation.

### 5.2 Modulation of matrix remodeling

To protect the articular cartilage from degradation, matrix remodeling enzymes such as MMPs and their tissue inhibitors (TIMPs), as well as “a disintegrin and metalloproteinase (with thrombospondin motifs)“ aggecanases (ADAM(TS)) move into focus. Upregulated and overactivated MMPs and ADAMTS play a central but insufficiently understood role in OA pathophysiology (Zeng et al., 2015; Yang et al., 2017; Mehana et al., 2019) with MMP-13 often being considered as key target (Li et al., 2017).

Resident connective tissue cells constantly remodel their ECM in response to various stimuli. Although relatively few studies have examined the role of MSCs in this process, available evidence suggests that they can modulate ECM degradation by releasing and regulating MMPs and TIMPs. For instance, human bone marrow MSCs were shown to inhibit the activity of MMP-2 and MMP-9 by secreting TIMP-2 and TIMP-1, respectively (Lozito and Tuan, 2011) - both MMPs playing a key role in mediating ECM degradation and remodeling. Importantly, MSCs increased TIMP-1 production in response to stimulation by inflammatory cytokines and 2% O2 hypoxia (Lozito and Tuan, 2011), pointing to a potential adaptive mechanism to inflammation-induced stress. Supporting this, an earlier study found that MSCs express MMP-2, -3, -10, -11, -13, -14 and TIMP-2 on both the mRNA and protein level, and inhibition of these MMPs using a broad-spectrum inhibitor significantly altered migration and proliferation abilities of MSCs (Kasper et al., 2007), reinforcing the functional relevance of MMP activity in MSC function.

Own studies in the context of tendon regeneration showed that MMP expression by MSCs, as well as the release of active MMPs, strongly depends on microenvironmental cues. In particular, fibrotic extracellular matrix triggered a downregulation of several MMPs and TIMP-2 (Doll et al., 2021; Burk et al., 2022) – which we primarily considered as a maladaptation in chronic tendon disease with erroneous ECM deposition, but which could also point towards mechanisms protecting ECM from degradation.

The hypothesis that MSCs – or their derivatives - provide beneficial effects in OA by modulating cartilage turnover is also supported by OA-related studies. In a canine *in vitro* model, adipose-derived MSCs did not only upregulate COL2A1, ACAN and SOX9, but also decreased MMP-13 gene expression in chondrocytes after LPS challenge. In synoviocytes, MMP-1, MMP-3 and MMP-13, as well as (transiently) ADAMTS-5 were downregulated by MSCs after the LPS challenge. The MSCs, in turn, upregulated hepatocyte growth factor (HGF), TSG-6 gene (TNFAIP6; Tumor necrosis factor-inducible gene 6), fibroblast growth factor (FGF)-2 and SOX9 gene expression in response to inflamed chondrocytes or synoviocytes. Some of these results differed between MSCs engineered to express PDGF or HO-1, or non-engineered MSCs. In a canine surgical *in vivo* model, MSC treatment entailed some clinically manifested benefits, with most improvement observed with PDFG-MSCs. Molecular analyses after *in vivo* treatment were not reported in this study (Oh et al., 2021).

Corresponding results were also observed with cell-free treatments. Human umbilical cord MSC-derived exosomes were used to treat either collagenase-induced or surgically induced OA in rats. In both models, articular cartilage preservation was significantly improved and pro-inflammatory cytokine concentrations in SF decreased. In supplementary *in vitro* experiments, the exosomes decreased MMP-13 and ADAMTS-5 and increased collagen II protein levels in IL-1β stimulated chondrocytes, yet again, no corresponding analyses after *in vivo* treatment were reported (Yang et al., 2024). A more extensive study in a surgical rat model revealed similar effects with conditioned medium from human bone marrow-derived MSCs, showing remarkable protection of cartilage and subchondral bone. Importantly, this was accompanied by stronger collagen II and aggrecan staining, decreased MMP-13 staining and increased TIMP-1 staining, the latter exceeding the sham control (Chen et al., 2019).

A further study, comparing the effects of human umbilical cord MSCs and their small EVs in a rat model where OA was induced by anterior cruciate ligament transection, showed that both treatments improved cartilage morphology and restored cartilage thickness. *In vitro*, small EVs were effectively internalized by chondrocytes and promoted their proliferation in a dose-dependent manner. *In vivo*, both treatments decreased the presence of MMP13 and ADAMTS5 but increased collagen II in the cartilage, and led to improved OARSI scores. As described in more detail below, in the same study, macrophage modulation was observed (Tang et al., 2021), possibly representing a mechanism involved in mediating the beneficial effects on the cartilage.

Similar findings were reported with exosomes harvested from human umbilical cord MSCs, which enhanced protein expression of collagen II and Sox9 in chondrocytes stimulated with IL-1β, and increased their ACAN, COL2 and SOX9, while reducing MMP13 and pro-inflammatory cytokine gene expression. Corresponding observations were made in a complementary surgical rat model, where exosome-treated joints displayed favorable outcomes with improved OARSI scores. Staining of type II and type X collagen revealed increased collagen II and reduced collagen X protein expression, and qRT-PCR showed effects on gene expression that mirrored the *in vitro* findings. All effects were further improved by MSC priming and encapsulation of the exosomes in methacrylated hyaluronic acid microspheres (Chen et al., 2025).

A further study in dogs, which can be considered of high translational value as dogs with naturally occurring bilateral elbow OA were included, showed corresponding changes within the synovial fluid after repeated injection of adipose-derived MSC conditioned medium obtained from a healthy donor dog. Namely, MMP-3 levels in SF decreased and TIMP-1 in SF increased from baseline over time, while there was also a decrease in pro-inflammatory mediators. Clinically, the range of motion significantly improved over time (Huňáková et al., 2020).

While these findings are promising and may provide explanations for MSC-driven cartilage ECM protection, it is to be acknowledged that there were also studies analyzing matrix modulatory enzymes which failed to demonstrate MMP down- and TIMP upregulation (Rahmadian et al., 2024). Furthermore, the network of MMP activation is complex and conclusions based on the downregulation of certain MMPs might be too simplistic. Finally, it is not yet clear whether the major contribution to modulating matrix remodeling comes from the MSCs directly, or whether these effects observed are indirect and mediated by other cells such as modulated macrophages- or the chondrocytes themselves.

### 5.3 Chondrocyte rescue

Complementing the observations on cartilage matrix preservation, some studies also revealed protective mechanisms of MSCs or their derivatives on chondrocytes. For instance, adipose-derived MSCs exerted anti-apoptotic effects on chondrocytes *in vitro*, while also reducing their expression of hypertrophic and fibrotic markers. This protective mechanism was mediated by HGF, the release of which by the adipose MSCs increased in co-culture with chondrocytes (Maumus et al., 2013). Similarly, as studied in rat *in vivo* models, exosomes or conditioned media from umbilical cord MSCs reduced chondrocyte apoptosis (Chen et al., 2019; Chen et al., 2025). The study using conditioned medium also reported an increase in autophagy, which was interpreted as equally beneficial (Chen et al., 2019).

However, other studies pointed towards decreased autophagy-while apoptosis was consistently inhibited-in response to MSC-derived exosome treatments: Exosomes derived from adipose-derived MSCs were found to downregulate IL-6, IL-1β, and TNF-α expression in IL-1β-treated chondrocytes. Beyond these anti-inflammatory effects, exosomal miR-93-5p inhibited both chondrocyte autophagy and apoptosis by targeting ADAMTS9, suggesting that the MSC-exosome/miR-93-5p/ADAMTS9 axis may represent a therapeutic target for OA (Li et al., 2023). Pointing out a further mechanistic link, the long non-coding RNA KLF3-AS1, delivered via MSC exosomes, was suggested to mediate reduction of chondrocyte injury and apoptosis by regulating miR-206/GIT1 axis (Liu et al., 2018). Further investigation into the role of MSC exosome-derived long non-coding RNA KLF3-AS1 revealed that IL-1β-damaged murine chondrocytes treated with human MSC exosomes showed increased KLF3-AS1 expression, reduced autophagy, decreased apoptosis, and improved viability. These effects were linked to the PI3K/Akt/mTOR pathway, activated via the YBX1 protein. When chondrocytes were treated with exosomes from KLF3-AS1-silenced MSCs, autophagy and apoptosis increased, while cell viability declined. KLF3-AS1 overexpression enhanced cell survival and reduced apoptosis, but these benefits disappeared when YBX1 was silenced (Wen et al., 2022).

Overall, there is evidence of direct chondrocyte protection by factors released by MSCs, resulting in decreased apoptosis and thus, representing a mechanism slowing OA progression. However, the effects on chondrocyte autophagy remain to be clarified along with further elucidating the role of autophagy in joint physiology versus OA pathogenesis. Here, possible mechanistic links between mitochondria (MT) and autophagy regulation (Rambold and Lippincott-Schwartz, 2011) might be important to consider. In this line, alongside regulatory RNAs delivered via exosomes, the means of direct chondrocyte protection could include the donation of mitochondria (MT) - an emerging mechanism that might have significance within the whole joint environment.

## 6 Mitochondrial transfer: an emerging rescue mechanism

Mitochondrial dysfunction is one of the earliest events in cartilage tissue following injury and contributes to cell death, ECM degradation and post-traumatic OA (Delco et al., 2018). As key regulators of reactive oxygen species (ROS), the dysfunction of mitochondria (MT) is closely linked to oxidative stress, a major cause of chondrocyte damage and cartilage degeneration (Yao et al., 2023; Wang et al., 2023). Oxidative stress resulting from MT dysfunction has been associated with increased MMP expression and activation of associated signaling pathways such as MAPK/ERK. This can suppress GAG and type II collagen synthesis while also promoting a shift towards fibrocartilaginous phenotypes (Rieder et al., 2018).

Inflammatory mediators such as interleukin-1 beta (IL-1β) and tumor necrosis factor-alpha (TNF-α) are also closely associated with MT dysfunction. Their upregulation has been shown to cause mitochondrial DNA (mtDNA) damage, reduction in adenosine triphosphate (ATP) synthesis, and impaired MT transcription (Qi et al., 2024; Zong et al., 2024) thereby creating a vicious cycle of cellular dysfunction. Of note, recent studies report that mtDNA in OA show signs of damage and mutation (Durán-Sotuela et al., 2024; Scotece et al., 2022). Such genomic instability can further impair mitochondrial function, leading to inadequate energy production.

This is important since chondrocytes require a sufficient supply of ATP to synthesize GAGs and Col II (Croucher et al., 2000), which is primarily generated through anaerobic glycolysis. Under glucose deprivation or glycolysis inhibition, chondrocytes transition to oxidative phosphorylation to maintain ATP production (Heywood et al., 2010) making MT functionality essential for preserving cartilage homeostasis under stress and inflammation. While chondrocytes can increase MT count via biogenesis, this requires time and an initial energy investment. Additionally, dysregulation of the PGC-1α/NRF-1 signaling axis, one of the key regulators of MT biogenesis, has been reported in OA-affected chondrocytes (Kim et al., 2021). Therefore, a lack of functional MT in chondrocytes is a likely key factor in the pathophysiology of OA.

As with chondrocytes, MT dysfunction in synoviocytes has been proposed to promote OA development (Li Y. et al., 2021). Hypoxia can cause loss of MT membrane potential in synoviocytes, leading to increased ROS production. This can activate the NF-κB pathway, elevating levels of pro-inflammatory cytokines such as CCL5, IL-1β, and IL-6 (Zhou et al., 2019). Additionally, folate deficiency has been shown to trigger synoviocyte apoptosis through MT complex II- and NADPH oxidase–induced ROS overproduction and calcium dysregulation, further reinforcing the proposed link between MT dysfunction in synoviocytes and OA progression (Hsu et al., 2016).

MSCs have been shown to transfer functional MT to injured cells as a rescue mechanism, which has been well-documented in several studies. It was first demonstrated in a co-culture of human MSCs and A549 ρ° cells, incapable of aerobic respiration through ethidium bromide-induced depletion of mtDNA. Rescued cells regained aerobic respiration, confirming that MT were successfully transferred from MSCs to the injured cells (Spees et al., 2006). Of relevance to OA, the first report describing MT transfer from MSCs to chondrocytes was published in 2019, using equine bone marrow-derived MSCs co-cultured with chondrocytes in which MT dysfunction had been induced either by a general inflammatory stimulus (IL-1β) or by specific MT stressors (oligomycin and rotenone). Authors verified MT transfer between the MSCs and chondrocytes following co-culture, with the highest transfer frequency occurring when chondrocytes were stimulated with rotenone and oligomycin (Bennett et al., 2019). In a subsequent report, the same group also described MT transfer to synoviocytes (Fahey et al., 2022).

Positive effects in chondrocytes following MT transfer from MSCs have also been reported. A study where rat bone marrow MSCs were co-cultured with chondrocytes from OA-affected joints reported that the activity of the MT respiratory chain complexes, MT membrane potential, and ATP content increased in chondrocytes post-transfer. The authors further suggested that MT transfer played a role in preventing mitochondrion-driven apoptosis in chondrocytes, with cell proliferation increasing by about 98% on the 7th day and total apoptosis rates dropping by about 55% (Wang et al., 2020). Moreover, MT transfer from MSCs to chondrocytes increased their DNA content and secretion of collagen II and proteoglycans (Wang et al., 2020; Korpershoek et al., 2022), suggesting that MT transfer from MSCs to OA chondrocytes not only supports MT function but may further contribute to improving chondrocyte activity and mitigating chondrocyte-related symptoms of OA. Collectively, these results indicate that MT transfer is an additional mode of action through which MSCs promote the regeneration of chondrocytes.

Following a different yet similar line of though, direct mitochondrial transplantation has been proposed as a novel strategy for restoring cellular bioenergetics in degenerative joint disease (Luo D. et al., 2024; Lee et al., 2022). In a recent study, chondrocytes, synovial macrophages, and synovial fibroblasts from human donors with OA were used to test the feasibility of transplanting MT from human umbilical cord-derived MSCs, with evidence of all cell types incorporating donor MT. The MT-treated chondrocytes displayed up to 21 upregulated and 6 downregulated genes. The upregulated genes were mostly associated with stress and inflammation, potentially demonstrating that MT transfer from MSCs does not only have metabolic benefits but can also upregulate pathways and proteins associated with OA-associated pathophysiological mechanisms.

To further study this, mice received unilateral intra-articular injections of type VII collagenase in knee joints to induce OA, and MT isolated from either 1 million or 200,000 MSCs were injected into the joint on day 7 and 14. Bone mineral density and bone surface-to-volume ratio both confirmed that MT injections had beneficial effects in all areas of the knee joint, while MT retention was observed only for roughly 1 day. Interestingly, beneficial effects were generally more evident with MT from lower MSC counts. Authors hypothesized that smaller doses could lead to more efficient MT uptake by the articular cells, while higher doses may cause oversaturation and less effective integration, potentially also leading to cellular stress (Vega-Letter et al., 2025). These findings suggest that MSC-derived MT could be a viable cell-free alternative to cell transplantation in OA, representing a further MSC derivative with a promising profile.

Still, MT alone are susceptible to damage during extraction and rely on endocytosis for cellular uptake. While more and more studies have been devoted to the development of engineered MT transplantations (Luo H. et al., 2024), delivering MT via their origin MSCs may be more effective, and comes with the additional benefits of the cell-based therapy. Early studies did not explore the mechanisms by which MSCs transfer MT, rather focusing on the effects observed in recipient cells after transfer. Subsequent research since identified several mechanisms through which MT transfer can occur between MSCs and cells involved in OA pathophysiology. MSCs can transfer MT via several mechanisms: tunneling nanotubes (TNTs), extracellular vesicles (EVs), gap junction–mediated delivery, and cell fusion (Velarde et al., 2022). We will highlight TNT- and EV-mediated mitochondrial delivery in this review.

### 6.1 Tunneling nanotube-vs. EV-mediated mitochondrial delivery

Tunneling nanotubes (TNTs) were first described in 2004, as highly sensitive nanotubular structures formed *de novo* between two or more cells (Rustom et al., 2004). These actin-containing formations were shown to facilitate the selective transfer of molecular cargos between cells. Specific markers for TNTs have yet to be identified, making morphological and functional properties, such as the presence of actin, still the primary criteria for TNT identification (Austefjord et al., 2014).

Although many aspects of this process are still unclear, we now understand that MSCs can use TNTs to directly transfer MT to target cells, both under physiological and pathological conditions. The first article to document TNT-mediated MT transfer between MSCs and chondrocytes used human bone marrow MSCs and cartilage obtained after debridement of focal cartilage lesions. Chondrocytes were treated with mitomycin C to induce senescence or TNF-α to mimic an inflammatory environment, and labelled MT were seen shuttling directly between MSCs and chondrocytes as well as between the chondrocytes themselves through broad actin-containing cell protrusions (Korpershoek et al., 2022). The same was observed in a study with immortalized chondrocytes challenged with tert-butyl hydroperoxide, resulting in MT depolarization and increased ROS production. Chondrocytes were then co-cultured with human bone marrow MSCs, and actin-positive filopodial extensions, containing labelled MT, were observed between the two cell types, with significantly more MT transfer events observed with the challenged chondrocytes (Irwin et al., 2024).

TNT-mediated MT transfer thus represents an emerging mechanism through which MSCs may exert their protective and regenerative effects on cells within the joint environment. Obviously, the transfer of MT via TNTs requires that MSCs and not their cell-free products are transplanted, and then attach within the joint. Given that MSCs do not readily attach to hyaline cartilage *in vivo*, this is an issue that questions the role of TNT-mediated MT transfer to chondrocytes *in vivo*, while MT transfer to synovial cells via TNTs could be a possible mechanism, although not yet directly evidenced.

Alternatively, MT transfer could take place via EVs, which might better allow to reach both chondrocytes and synoviocytes *in vivo*. When studying MT transfer from different species’ bone marrow-derived MSCs to injured chondrocytes using different *in vitro* models, observations pointed to modes of MT donation beyond TNT-mediated transfer (Fahey et al., 2022). The same group confirmed that functional human bone marrow MSC-derived EVs could be taken up by chondrocytes treated with rotenone and antimycin. Based on their size, EVs were divided into three clusters: small (5–10 nm in diameter), medium (100–1,000 nm) and much larger vesicles (5,000–10000 nm). An average of 71% of intact EVs contained MT, with larger EVs being the more likely to contain them. Of these, an average of 31% were functional MT, as demonstrated by polarization staining of the MT in the EVs. However, the uptake of EVs by the chondrocytes was relatively low, requiring future optimization of experimental conditions (Thomas et al., 2022). Building on this concept, a recent study demonstrated that MT-rich EVs derived from human synovial fluid-derived MSCs could transfer MT to chondrocytes stressed with IL-1β. Interestingly, levels of oxidative stress-associated mediators γH2AX, ROS, and MitoSOX were lower in both stressed and untreated chondrocytes when incubated with MSC-derived EVs (Li et al., 2025).

Irrespective of the transfer route, the consistent observation of beneficial outcomes on recipient cells after MT transfer across several studies is highly encouraging. However, further research is necessary to identify the factors that regulate TNT formation vs. MT uptake in EVs, as well as on the circumstances under which MSCs use different means of MT transfer.

### 6.2 Mitochondrial transfer regulation and induction mechanisms

The transmembrane protein connexin 43 (Cx43) helps create pores in the cell membrane that either communicate with the extracellular environment or form gap junctions with adjacent cells (Irwin et al., 2024). Cx43 is considered as one of the key regulators of MT transfer between various cell types, facilitating both EV- and TNT-mediated MT transfer (Yao et al., 2018; Yang et al., 2023). Manipulation of Cx43 expression, either through overexpression or silencing, affected TNT formation and MT transfer between induced pluripotent stem cell-derived MSCs and epithelial cells (Yao et al., 2018). Similarly, enhanced Cx43-mediated gap junction communication was reported to increase TNT formation and release of microvesicles from MSCs (Islam et al., 2012). With respect to cartilage, both pharmacologic (carbenoxolone disodium) and Cx43-mimetic peptide (Gap 27)-mediated inhibition of gap junctions were found to decrease MT transfer between MSCs and murine chondrocytes (Fahey et al., 2022). Notably, chondrocyte Cx43 expression is increased in OA (Mayan et al., 2013) and when stressed with IL-1ß (Tonon & D'Andrea, 2000).

Gap junction alpha-1 protein (GJA1), the gene encoding Cx43, has been shown to undergo alternative translation into several isoforms (Smyth and Shaw, 2013), with the GJA1-20k isoform being particularly relevant to MT transfer due to its role in MT trafficking along microtubules (Fu et al., 2017), and in actin recruitment for cell trafficking pathways (Basheer et al., 2017). Overexpression of GJA1-20k has been reported to enhance MT transfer from astrocytes to neurons *in vitro* (Ren et al., 2022). The role of Cx43 in chondrocytes was validated using siRNA to knockdown Cx43 expression in MSCs prior to co-culture, with a significant reduction in both full-length Cx43 and the isoform GJA1-20k being evident. Chondrocytes co-cultured with GJA1 siRNA-treated MSCs also showed fewer MT transfer events compared to those co-cultured with control MSCs. However, MT transfer was not entirely suppressed, indicating the possible involvement of additional, Cx43-independent transfer mechanisms in chondrocytes (Irwin et al., 2024).

Interestingly, MT transport was also documented from chondrocytes to MSCs in one of the above-mentioned studies (Koperpershoek et al., 2022). The authors hypothesized that transfer of defective MT from chondrocytes to MSCs could be a way for chondrocytes to signal cellular stress to MSCs, while depolarized MT might also be fused with MT in MSCs to rescue their metabolic state. Additionally, defective MT could be presented to MSCs to be disposed of, a process also known as transmitophagy (Davis et al., 2014), while also preventing further damage from oxidative stress in the chondrocytes.

### 6.3 Possible induction mechanisms and effects on recipient cells

It is also important to note that the triggers of intercellular MT transfer, and why MSCs use four different mechanisms to do so, remain largely unknown. Theories suggest that besides the presence of damaged MT, oxidative stress induced by MT-dysfunction in recipient cells may send cues for MSCs to initiate MT transfer (Islam et al., 2012; Ahmad et al., 2013). It has been observed that mtDNA from injured cells can be engulfed by MSCs, subsequently triggering their cytoprotective functions and enhancing MT biogenesis through retrograde signaling, preparing the MSCs for MT donation (Mahrouf-Yorgov et al., 2017). On the other hand, under stress conditions, MSCs have been shown to release depolarized mitochondria via EVs, transferring them to macrophages to outsource mitophagy for self-protection (Phinney et al., 2015).

Bidirectional MT transfer is a compelling mechanism through which MSCs can exert protective and regenerative effects on resident cells in OA. Accumulating evidence suggests the partial reversibility of oxidative stress–induced tissue degeneration highlights mitochondrial function as a viable therapeutic target (He et al., 2025). As summarized by a recent review, MT transfer has shown beneficial effects in recipient cells post-MT transfer in several disease contexts, including the restauration of oxidative phosphorylation, reduction of ROS accumulation and anti-apoptotic effects (He et al., 2025). Reports also indicate improved redox balance, energy status, and metabolic homeostasis in osteoarthritic chondrocytes, thereby preserving cartilage integrity (Court et al., 2024). Despite significant progress in elucidating these processes, the precise upstream mechanisms governing the initiation of MT transfer, as well as factors determining the chosen transfer route and direction of transfer remain incompletely understood. Future research aimed at optimizing and modulating MT transfer offers novel opportunities for MSC-based therapies, targeting mitochondrial dysfunction in OA-affected chondrocytes and synoviocytes, thereby supporting cartilage preservation and repair.

## 7 Conclusion and prospects

New insights into MSC-mediated effects in OA are emerging rapidly, yet the intricate, context-dependent interplay among synovial, cartilage, and immune cells still obscures which mechanisms could ultimately drive disease modification. What is increasingly clear is that the joint must be treated as an integrated organ, with interventions aimed at restoring system-level homeostasis rather than singular tissue repair. Accordingly, prioritizing pan-cellular, convergence mechanisms-such as mitochondrial transfer, alongside EV-mediated signaling and macrophage modulation-offers a rational path to restore joint homeostasis.
